# An Overview of Biological and Computational Methods for Designing Mechanism-Informed Anti-biofilm Agents

**DOI:** 10.3389/fmicb.2021.640787

**Published:** 2021-04-13

**Authors:** Andy Y. An, Ka-Yee Grace Choi, Arjun S. Baghela, Robert E. W. Hancock

**Affiliations:** Centre for Microbial Diseases and Immunity Research, University of British Columbia, Vancouver, BC, Canada

**Keywords:** biofilms, antibiotic resistance, anti-biofilm agents, systems biology, virtual screening, machine learning, biofilm models, organoids

## Abstract

Bacterial biofilms are complex and highly antibiotic-resistant aggregates of microbes that form on surfaces in the environment and body including medical devices. They are key contributors to the growing antibiotic resistance crisis and account for two-thirds of all infections. Thus, there is a critical need to develop anti-biofilm specific therapeutics. Here we discuss mechanisms of biofilm formation, current anti-biofilm agents, and strategies for developing, discovering, and testing new anti-biofilm agents. Biofilm formation involves many factors and is broadly regulated by the stringent response, quorum sensing, and c-di-GMP signaling, processes that have been targeted by anti-biofilm agents. Developing new anti-biofilm agents requires a comprehensive systems-level understanding of these mechanisms, as well as the discovery of new mechanisms. This can be accomplished through omics approaches such as transcriptomics, metabolomics, and proteomics, which can also be integrated to better understand biofilm biology. Guided by mechanistic understanding, *in silico* techniques such as virtual screening and machine learning can discover small molecules that can inhibit key biofilm regulators. To increase the likelihood that these candidate agents selected from *in silico* approaches are efficacious in humans, they must be tested in biologically relevant biofilm models. We discuss the benefits and drawbacks of *in vitro* and *in vivo* biofilm models and highlight organoids as a new biofilm model. This review offers a comprehensive guide of current and future biological and computational approaches of anti-biofilm therapeutic discovery for investigators to utilize to combat the antibiotic resistance crisis.

## Introduction

Bacterial biofilms are complex three-dimensional (3D) aggregates of microbes on surfaces including body surfaces, medical devices, and wounds. The National Institutes of Health estimate that biofilms are involved in 65-80% of all microbial infections and 80-90% of all chronic infections, making biofilms a significant healthcare issue (Attinger and Wolcott, 2012; Römling and Balsalobre, 2012; Jamal et al., 2018). Biofilm growth is an adaptive growth state and critically, biofilm aggregates are highly (adaptively) antibiotic resistant when compared to the same bacteria in their free-floating planktonic form (Verderosa et al., 2019). With the growing antibiotic crisis fueled by antibiotic overuse and potentially accelerated by recent events such as COVID-19 (Strathdee et al., 2020), understanding biofilm formation, combatting antibiotic resistance, and developing new anti-biofilm agents are key priorities in health care.

Despite this necessity and priority, there are currently no approved anti-biofilm agents. Of the 82 registered clinical trials with known status (recruiting, active, completed, or terminated) on clinicaltrials.gov involving biofilm treatment or measurement, 25 involve testing a drug for anti-biofilm effects, mainly against oral biofilms. Most of these studies apply general antiseptics (e.g., chlorhexidine) or antibiotics (e.g., cefazolin), which are not biofilm-specific. However, there are currently two ongoing trials that are assessing anti-biofilm specific agents. The first is using nitric oxide, a known regulator for biofilms (Barraud et al., 2006), against chronic rhinosinusitis (Phase 2, NCT04163978). The other is using TRL1068, a human monoclonal antibody against the bacterial protein DNABII (which stabilizes DNA in the extracellular matrix of biofilms) (Xiong et al., 2017), against prosthetic joint infections (Phase 1, NCT04763759). Despite years of research, the fact that there are only two anti-biofilm candidates in the pipeline, and none approved, attests to the difficulty of creating anti-biofilm agents. This is likely due to a combination of a lack of priority given to this class of drugs, inaccurate biofilm models (that show efficacy *in vitro* and/or *in vivo* but not in humans) and an inadequate understanding of biofilm formation.

**FIGURE 1:**
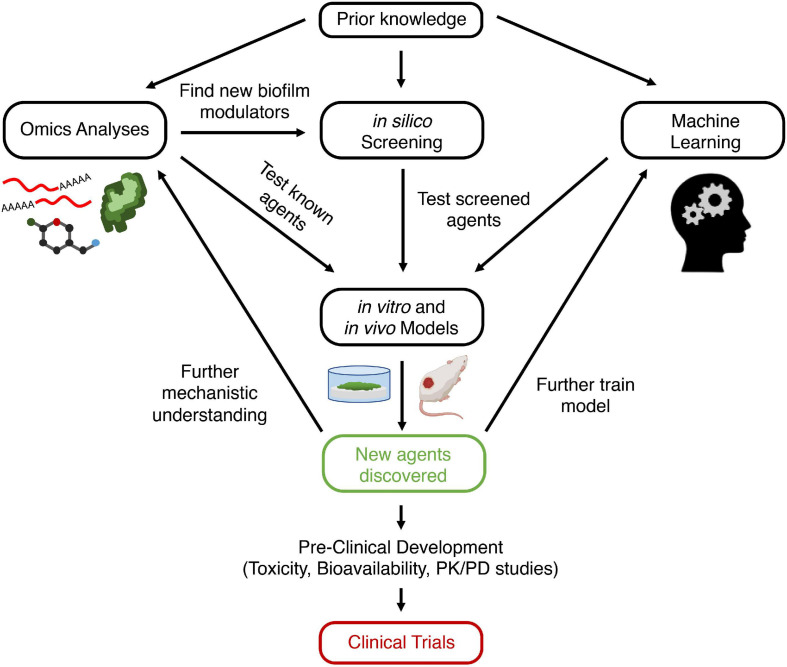
Schematic view of approach for discovering new anti-biofilm agents. Prior knowledge leads to hypothesis generation and exploration of biofilm formation mechanisms. This can be probed using omics analyses, which can lead to the discovery of new anti-biofilm targets (genes, proteins, metabolites). Modulators of these targets (e.g., inhibitors of quorum sensing receptors) are screened directly using *in vitro* or *in vivo* models. Alternatively, *in silico* screening can be performed first on databases of compounds to identify those that bind to and modulate biofilm regulating proteins, which can then be validated with *in vitro* or *in vivo* models. Conversely, databases of known anti-biofilm agents can be used to train a machine learning model. The algorithm can then screen for putative anti-biofilm agents that are validated with *in vitro* and *in vivo* models. Finally, new agents that are discovered to be effective can undergo preclinical studies and then be entered into clinical trials and ultimately be used for human disease. In addition, these new agents can lead to further understanding of biofilm mechanisms, as well as providing additional data for optimization of machine learning models. Created with BioRender.com. PK, pharmacokinetics; PD, pharmacodynamics.

To accelerate discovery of novel anti-biofilm agents, we must leverage newer and more biologically relevant models, as well as new sequencing and computational technologies to better understand biofilm formation. Thus, in this review, we begin by describing current literature on biofilm formation and resistance, as well as the mechanisms of some existing anti-biofilm agents. We then describe how to employ a set of biological and computational methods to develop novel anti-biofilm agents to be used as a guide for investigators interested in anti-biofilm agent discovery. Most studies exploring biofilm mechanisms rely on omics studies, such as transcriptomics and proteomics, to uncover new genetic and protein targets for novel anti-biofilm agents to modulate. *In silico* screening can be used to screen for molecules from large databases that bind to and modulate these targets. Another approach is machine learning, in which algorithms are repetitively employed to predict the anti-biofilm activity of a molecule. Candidate molecules identified using machine learning or *in silico* screening can then be synthesized and validated in a variety of biological models, including biofilms grown in microtiter plates, flow cells, animal models, and human organoids. Successful candidates can then strengthen knowledge of biofilm formation mechanisms, further train machine learning algorithms, and ideally transition to clinical trials for human usage. Integrating multiple modalities of both lab and computational science can give investigators a better chance at developing a successful anti-biofilm agent (Figure 1).

## The Clinical Relevance of Biofilms

Biofilms can colonize biological or nonbiological surfaces, putting all patients, but especially the immunocompromised, surgical patients, individuals with major injuries or burns, and patients with implanted devices, at a high risk of developing biofilm infections. Critically, biofilms are associated with many or most chronic infections and are often associated with chronic inflammation, pain, and tissue damage. Biofilm-associated disease can affect virtually any organ system, most notably the cardiovascular (e.g., endocarditis), respiratory (e.g., cystic fibrosis), urinary (e.g., urinary tract infections), and oral (e.g., periodontitis) systems (Vestby et al., 2020). Implanted medical devices, such as catheters, stents, prosthetic heart valves, pacemakers, and artificial joints or limbs, are also common sites of biofilm formation (Bryers, 2008). Furthermore, planktonic bacteria can detach from the biofilm to spread throughout the body, causing bacteremia, colonizing other organ systems, forming thromboemboli, or triggering a septic episode (Fleming and Rumbaugh, 2018). Bacteria in biofilms are notoriously difficult to remove from abiotic surfaces such as door handles, beds, taps, showers, and other high-touch surfaces in the hospital setting, with such biofilms frequently containing multiple species of drug-resistant bacteria (Vickery et al., 2012). Persistence also occurs on biotic surfaces with chronic wounds. Biofilms colonize 60% of ulcers in diabetic patients, which can lead to limb amputation (James et al., 2008), and cause major problems in chronic rhinosinusitis (Karunasagar et al., 2018). The prevalence and persistence of biofilms can be attributed to a biofilm’s ability to resist agents that would normally act against bacteria, including the host immune response and antibiotic treatment. Generally speaking, antibiotics have dramatically decreased mortality from infectious diseases. However, antibiotics have been almost exclusively developed and evaluated for efficacy against planktonic bacteria and are relatively ineffective against biofilms. Decades of research have sought an understanding of the biofilm processes that cause this resistance, with only moderate insights. Importantly, we need to understand unique biofilm biology in order to develop new anti-biofilm agents to specifically target biofilm processes and treat chronic infections.

Resistance describes a bacterium’s ability to grow despite antibiotic treatment and is usually measured by the minimum inhibitory concentration (MIC, the lowest concentration of an antibiotic that inhibits bacterial growth). Biofilm resistance to antibiotics reflects the unique growth state of biofilms. First and foremost, biofilms undergo transcriptional reprogramming to the state that is intended to resist stress (de la Fuente-Núñez et al., 2013; Taylor et al., 2014). Since antibiotics are one type of stressor, it can be anticipated that alterations in the expression of genes in the resistome (encompassing all potential resistance mechanisms in any given bacterium) lead to decreased susceptibility, and that this likely involves multiple genes, as shown for other complex adaptive growth states such as swarming and surfing motility in *Pseudomonas aeruginosa* (Sun et al., 2018; Coleman et al., 2020). Our own current research is leading us to believe that this is also true for biofilms and there is considerable evidence supporting this perspective (Liao et al., 2013). This type of resistance is termed adaptive resistance or tolerance since it reverts as soon as the organisms are no longer growing as biofilms. It seems likely that at least some of the mechanisms involved are similar to those involved in resistance in planktonic cells (Hall and Mah, 2017) but exacerbated by the biofilm growth state, although unique regulatory genes and effectors might be involved. Evaluation of the resistome in planktonic cells has shown that mutations in numerous genes can lead to resistance to any given antibiotic (Breidenstein et al., 2008; Schurek et al., 2008; Gallagher et al., 2011; Coleman et al., 2020).

Other aspects of the biofilm growth state include increased cellular proximity, which has been shown to enhance horizontal gene transfer in biofilms compared to planktonic populations, resulting in faster acquisition of genetically resistant mutants in a biofilm (Molin and Tolker-Nielsen, 2003). In addition, the frequency of mutations appears to be enhanced in biofilms, perhaps due to increased oxidative stress (Driffield et al., 2008). Additional resistance of biofilms is conferred by the extracellular matrix, consisting of species-specific polysaccharides and proteins as well as extracellular DNA (Ciofu and Tolker-Nielsen, 2019). As a gel that loosely encapsulates and holds together the biofilm, the matrix may decrease the penetrance of certain but not all antibiotics (Singh et al., 2016). For example, the positively charged antibiotic tobramycin was sequestered by the matrix in *P. aeruginosa* biofilms, while the neutral antibiotic ciprofloxacin was able to penetrate (Tseng et al., 2013). Finally, nutrient gradients in a biofilm result in hypoxic regions within the biofilm, leading to less metabolically active bacteria (Stewart et al., 2016). These dormant bacteria can survive but not necessarily grow in the presence of antibiotics, a form of tolerance (Lebeaux et al., 2014). Antibiotics generally target active cells by inhibiting biosynthetic pathways; therefore, they are largely ineffective against dormant cells (Ciofu and Tolker-Nielsen, 2019). Thus, while active bacteria on the surface of biofilms may be eradicated by antibiotics, dormant persister cells are able to survive and become active once the antibiotic regimen is concluded, resulting in chronic infections (Høiby et al., 2010; Lebeaux et al., 2014). With all these mechanisms involved, biofilms are up to 1000-fold more resistant to multiple antibiotics than planktonic bacteria (Ceri et al., 1999).

Since resistance relies on the biofilm growth state, targeting biofilms, either by inhibiting formation or stimulating the dispersal of mature biofilms, is an obvious path to overcoming resistance of biofilms to antibiotic therapies. Intriguingly, there are demonstrations that biofilm inhibitors can act synergistically with conventional antibiotics (de la Fuente-Núñez et al., 2015). Unfortunately, there is not a single approved treatment for biofilms presently, so this is an area that deserves attention. Critically, the first step to developing these therapies is understanding the mechanisms of biofilm formation.

## Biofilm Formation Mechanisms and Existing Therapies That Target Them

Biofilms start as individual planktonic bacteria that can reversibly attach to surfaces. This can then lead to changes in gene expression that trigger irreversible binding, in part driven by the expression of particular adhesins. Concurrently, bacteria begin to secrete matrix components and the biofilm matures into a multilayer structure (Armbruster and Parsek, 2018). This complex process is regulated by multiple processes that have been extensively reviewed previously (Rabin et al., 2015; Tolker-Nielsen, 2015; Roy et al., 2018). Here we will highlight three major regulatory networks that appear to be somewhat conserved and are attractive targets for novel anti-biofilm agents, namely the stringent response, quorum sensing, and cyclic di-guanosine monophosphate (c-di-GMP) signaling.

### The Stringent Response

All bacteria produce the nucleotide second-messengers/alarmones guanosine tetraphosphate and pentaphosphate [collectively (p)ppGpp] as part of the stringent stress response. Synthesis of these molecules is induced when a bacterial population is undergoing diverse nutritional stresses including limitations of carbon sources, amino acids, fatty acids, iron, and phosphate, but it is also clear that these molecules have important functions under normal growth conditions (Pletzer et al., 2020). Diverse enzymes mediate (p)ppGpp metabolism including ribosome-associated RelA synthase and SpoT in Gram negative bacteria and the bi-functional enzyme Rsh in Gram positives. The accumulation of (p)ppGpp results in a reprogramming of bacterial cells to adapt to nutrient deprivation, including decreasing macromolecular synthesis while upregulating stress accommodating pathways (Ross et al., 2016; Pletzer et al., 2020). The stringent response regulates biofilm formation in multiple Gram positive and Gram negative species (Balzer and McLean, 2002; He et al., 2012; de la Fuente-Núñez et al., 2014; Azriel et al., 2016; Liu et al., 2017). Mutants with deletions in (p)ppGpp synthases in *P. aeruginosa*, *S. aureus*, *E. coli*, *Salmonella*, *Listeria monocytogenes*, and *Enterococcus faecalis*, were either unable to form biofilms or formed poorly structured biofilms (Taylor et al., 2002; Chávez de Paz et al., 2012; de la Fuente-Núñez et al., 2014).

Due to its ubiquity in bacterial species and necessity for successful biofilm formation, (p)ppGpp is an excellent target for anti-biofilm therapies. Specific cationic amphipathic peptides, related to antimicrobial and host defense peptides, have preferential broad spectrum anti-biofilm activity which is mediated by binding directly to (p)ppGpp, marking it for degradation (de la Fuente-Núñez et al., 2014, 2015). While this class of peptides can have a variety of functions, including host immune system modulation, anti-inflammatory activity, wound healing, and direct antibacterial activity vs. planktonic bacteria (Haney et al., 2015), specific anti-biofilm activity was first observed with sub-inhibitory concentrations of LL-37 (Overhage et al., 2008) and subsequently with synthetic peptides such as IDR-1018 and the D-enantiomeric peptide DJK-5 (de la Fuente-Núñez et al., 2014, 2015). Excitingly, these peptides exhibit very broad spectrum activity against biofilms formed from all of the major antibiotic resistant pathogens in our society (collectively called the ESKAPE pathogens) (de la Fuente-Núñez et al., 2014, 2015; Pletzer et al., 2018), work against preformed biofilms and multispecies biofilms such as oral biofilms (Zhang et al., 2016; Wang et al., 2017), demonstrate synergy with conventional antibiotics *in vitro* (de la Fuente-Núñez et al., 2015) and *in vivo* (Pletzer et al., 2018), and work in several animal models. These peptides act in part against the stringent stress response and, in a murine abscess model, they also inhibit the transcription of (p)ppGpp-metabolizing enzyme SpoT, while it was proposed that there might be other or additional mechanisms explaining their action against biofilms (Pletzer et al., 2017; Salzer et al., 2020). Design features that discriminate such anti-biofilm peptides are different from those mediating activity against planktonic cells (de la Fuente-Núñez et al., 2014; Haney et al., 2018a). Thus, antibiofilm peptides are an attractive class of molecules that can be further optimized through rational design (see below) or synthesis of peptidomimetics (Gomes Von Borowski et al., 2018).

Instead of directly targeting (p)ppGpp, another method of stringent response modulation is through inhibition of (p)ppGpp synthetases, which is still a relatively unexplored field. The majority of known inhibitors are (p)ppGpp analogs such as Relacin (Wexselblatt et al., 2012) although these analogs have multiple off-target effects and low binding affinities (Wexselblatt et al., 2013). Given the recent characterization of synthetase structures, such as from *E. coli* (RelA) and *S. aureus* (RelP, RelQ), it will now be possible to use *in silico* screening methods to identify new inhibitors (Hall et al., 2020).

### Quorum Sensing

Quorum sensing (QS) refers to the ability of bacteria within a population, such as a biofilm, to regulate gene expression based on cell density. QS is facilitated through small signaling molecules generated by bacteria that self-regulate their own expression through a positive feedback loop and are thus termed auto-inducers (Fetzner, 2015). Gram positive bacteria most commonly use auto-inducing cyclic peptides as auto-inducers, while Gram negative bacteria primarily use N-acyl homo-serine lactones (AHLs), quinolones, and fatty acids (Heeb et al., 2011; Schuster et al., 2013; Monnet et al., 2016; Zhou et al., 2017). Both Gram positive and negative bacteria can also use a furanosyl borate diester called autoinducer-2, suggesting the possibility of cross-talk between different species of bacteria in a community (De Keersmaecker et al., 2006). Auto-inducers are produced and at a sufficient extracellular concentration are taken up and bind to their cognate receptors/transcription factors to exert their functions, including upregulating genes for virulence factors, antibiotic resistance, and biofilm formation (Liang et al., 2014).

Interfering with QS does not prevent biofilm formation but can have strong effects. For example, a *P. aeruginosa* mutant with a *lasI* deletion (which cannot produce the AHL 3-oxo-C12-HSL) has slower biofilm formation and flatter biofilms (Shih and Huang, 2002), while addition of 3-oxo-C12-HSL to this mutant allowed formation of biofilms structurally similar to wild type (Davies et al., 1998). Similarly, mutations in QS genes in *Burkholderia cepacia* and *Aeromonas hydrophila* also resulted in impaired biofilm formation (Huber et al., 2001; Lynch et al., 2002). QS interference can also result in biofilms that are more susceptible to antibiotic treatment and host immune responses. *P. aeruginosa* biofilms that were treated with QS inhibitors C-30 and C-56 furanones had increased sensitivity to tobramycin, while *lasI* mutants were more susceptible to kanamycin (Hentzer et al., 2002; Shih and Huang, 2002). *P. aeruginosa* with deletions in the Las and Rhl QS systems (*lasR, rhlA*, and *rhlR*) formed biofilms that were cleared more efficiently by polymorphonuclear cells compared to wild-type (Bjarnsholt et al., 2005; Gennip et al., 2009). Thus, therapies that inhibit QS (termed “quorum quenchers”) represent a potential therapy targeting biofilms.

Quorum quenching can be divided into four mechanisms: (i) inhibiting auto-inducers from binding to their receptors, such as using halogenated furanones (Hentzer et al., 2002; Hentzer, 2003); (ii) decreasing production of auto-inducers by targeting their synthases, such as MvfR in *P. aeruginosa* (Starkey et al., 2014; Maura and Rahme, 2017); (iii) sequestering auto-inducers using cyclodextrans or antibodies (Park et al., 2007; Morohoshi et al., 2013); and (iv) degradation of auto-inducers using enzymes such as lactonases (Rémy et al., 2018). Most quorum quenchers that inhibit auto-inducer binding are derived from natural products (Rémy et al., 2018). However, QS systems are different across species, and generating broad-spectrum quorum quenchers might not be possible, and even different species sharing the same QS system may behave differently to a particular quorum quencher (Galloway et al., 2011). In the future, quorum quenchers might be used with conventional antibiotics since some quorum quenchers make biofilms more sensitive to conventional antibiotic use. However, while promising *in vitro* data is widely available, no quorum quenchers have been successfully tested in clinical trials for biofilm treatment (Hansen et al., 2005). It is also important to realize that quorum quenchers should be used only for specific species, since in some bacteria, such as *Vibrio cholerae*, QS actually represses biofilm formation to promote dispersal under high-density conditions. Thus, using a quorum quencher in this case could result in further aggregation of biofilms (Waters et al., 2014).

### c-di-GMP Signaling

Signaling through c-di-GMP, a second-messenger molecule, is a significant player in controlling the transition from a motile to sessile (biofilm) lifestyle (Jenal et al., 2017). In most cases, high levels of c-di-GMP bind to downstream effectors such as transcriptional regulators, mRNA riboswitches, and protein adaptors to, among others, reduce the expression of motility (e.g., flagellar) genes and increase the expression of genes required for biofilm formation (Jenal et al., 2017). For example, in *P. aeruginosa*, higher c-di-GMP results in the increased expression of matrix components including adhesins (CdrA) and polysaccharides (Pel, Psl) (Borlee et al., 2010; Ha and O’Toole, 2015). The levels of c-di-GMP are controlled by multiple synthetic diguanylate cyclases and degradative phosphodiesterases, and both enzymes are heavily regulated by environmental cues, such as pathways regulated through QS (Srivastava and Waters, 2012). Thus, inhibiting diguanylate cyclases or activating phosphodiesterases to reduce the level of c-di-GMP may be another method of countering biofilms.

The fact that bacteria often have more than a dozen diguanylate cyclases and phosphodiesterases, which vary substantially between organisms, makes the possibility of drug development somewhat intimidating. However, various classes of diguanylate cyclases inhibitors have been developed. These include GTP or c-di-GMP analogs, which inhibit diguanylate cyclases in the active site and an allosteric site, respectively (Cho et al., 2020). Small molecule inhibitors of diguanylate cyclases have also been discovered using high-throughput *in vitro* and *in silico* screening (Cho et al., 2020), although activities tend to be modest. Stimulating activity of phosphodiesterases has been accomplished using nitric oxide donors such as sodium nitroprusside, leading to dispersal of *P. aeruginosa* biofilms (Barraud et al., 2006). Much like QS and the stringent response, the availability of structures of the specific proteins involved in regulating c-di-GMP pathways provides the necessary data to perform virtual screening for new inhibitors, as discussed below. A new avenue that works on a common property of bacteria is c-di-GMP sequestration using rationally designed peptides that mimic the structure of an effector protein to which c-di-GMP binds; such peptides have been shown to inhibit *P. aeruginosa* biofilm formation (Hee et al., 2020).

## Bioinformatic Approaches to Understand Mechanism for Novel Anti-Biofilm Agents

**FIGURE 2:**
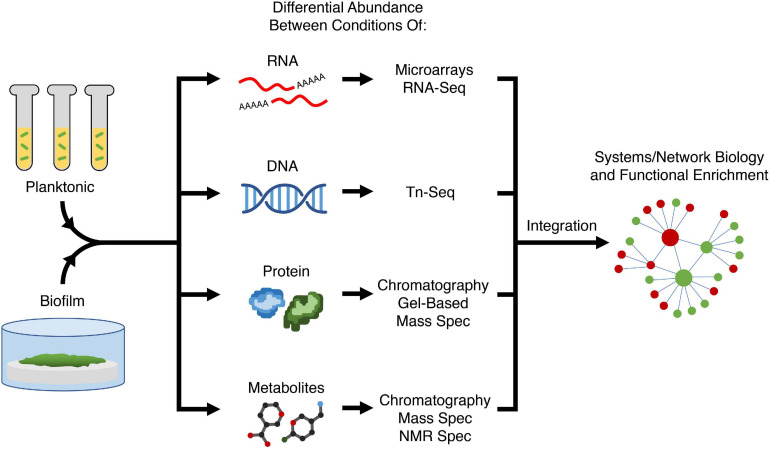
Variety of omics approaches for elucidating biofilm mechanisms. Two or more conditions (e.g., planktonic vs. biofilm growth, presence or absence of an anti-biofilm agent, different biofilm substrates) are compared in terms of abundance of most biological molecules in the cell; with different methods being used to assess RNAs, transposon-insertions, proteins, and metabolites. Using systems or network biology, data from different modalities can be integrated to perform functional enrichment of genes and pathways required for biofilm formation to generate a holistic mechanistic view of biofilm formation. Tn-Seq, transposon insertion sequencing; Spec, spectroscopy; NMR, nuclear magnetic resonance.

A large proportion of current anti-biofilm agents have been developed by specifically targeting a process understood to regulate biofilm formation. Therefore, to develop new anti-biofilm agents, better understanding of biofilm formation is required to find new targets. Conversely, there are also existing anti-biofilm agents for which the precise mechanism of action is still unclear, and therefore understanding how these agents act on biofilms can provide new avenues and/or targets for modulation by new agents. Omics approaches such as transcriptomics, genomics, proteomics, and metabolomics are key to uncovering target genes, pathways, and processes required for biofilm formation. In general, each approach looks for differential abundance of biological molecules (nucleic acid, proteins, metabolites) between conditions. By comparing molecular changes between different conditions (e.g., bacteria in biofilms vs. planktonic growth, mutant vs. wild-type strains, ± an anti-biofilm agent), one can hypothesize that the observed changes reflect the condition or treatment (Figure 2) and potentially reveal details about mechanisms and potential causes of resistance. These omics approaches yield a vast amount of data and thus a systems biology approach is needed for analysis. A common technique to group genes together is through pathway enrichment, using databases such as Kyoto Encyclopedia of Genes and Genomes (KEGG) or MetaCyc, and functional enrichment using gene ontology (GO) terms, in order to determine which pathways and functions are dysregulated and therefore potential targets for modulation (Kanehisa and Goto, 2000; Karp et al., 2002; Gene Ontology and Consortium, 2015).

### Transcriptomics

RNA-Seq is a high-throughput technology employed to measure gene regulation and expression. Numerous studies have appeared in the literature using RNA-Seq (or its precursor microarray technology) to identify differentially expressed genes between planktonic and biofilm lifestyles for a variety of bacterial species including *P. aeruginosa* (Dötsch et al., 2012), *Klebsiella pneumoniae* (Guilhen et al., 2016), *Campylobacter jejuni* (Tram et al., 2020), *Bacillus licheniformis* (Sadiq et al., 2019), revealing that biofilm formation leads to hundreds of dysregulated genes (Amador et al., 2018). For example, RNA-Seq allowed for the identification of transcriptomic signatures specific to planktonic, biofilm, and biofilm-dispersed *K. pneumoniae* cells, highlighting underlying mechanisms involved in each bacterial lifestyle (Guilhen et al., 2016). RNA-Seq can also be used to study the effect of antibiotics and potential anti-biofilm agents on biofilm formation (Tan et al., 2015; Liu et al., 2018). Recently, Wu et al. (2020) probed the anti-biofilm effects of exopolysaccharide EPS273 on *P. aeruginosa* using RNA-Seq and found that EPS273 might mediate its effects by downregulating expression of genes in the PhoP-PhoQ two-component system and QS systems LasI/LasR and RhII/RhIR, which are involved in biofilm formation. These studies elucidated new pathways that can be targeted by novel therapies. As RNA-Seq costs decrease, technical methods improve, and better *in vitro* and *in vivo* models are developed for biofilm analysis, it is also now possible to perform dual RNA-Seq of both the host and pathogen to interrogate host-pathogen interactions (Westermann et al., 2017). To illustrate the potential applications of RNA-Seq for biofilm studies, we highlight two recent studies on complex adaptive lifestyles from our lab that employed RNA-Seq technologies.

Coleman et al. (2020) aimed to identify dysregulated genes that allowed *P. aeruginosa* to resist tobramycin while in the swarming state. Swarming motility is a coordinated surface-associated movement that occurs under conditions that mimic the surface of the human lung and has been proposed to allow for rapid colonization leading to biofilm formation in the cystic fibrosis lung. This adaptive growth state, like biofilm formation, leads to resistance to multiple antibiotics. RNA-Seq identified 29% (1581) of genes that were differentially expressed (DE) in swarming compared to swimming motility (behavior of bacteria in aqueous environments). From these, 26 DE genes were identified that were proven to be involved in swarming mediated resistance to tobramycin, demonstrating that adaptive resistance was multigenic. For example, genes in the *wbp* operon involved in lipopolysaccharide synthesis were downregulated, indicating a new role in lipopolysaccharide alteration for adaptive tobramycin resistance. Thus, this approach highlights the mechanistic changes that occur to promote tobramycin resistance in swarming *P. aeruginosa*. A further 224 genes were DE between tobramycin-treated and untreated swarming *P. aeruginosa* and many downregulated genes were identified using GO as virulence factors and QS regulators, indicating while tobramycin might not kill swarming *P. aeruginosa*, it may still have clinical benefits in dampening virulence. A notable upregulated gene in swarming cells treated with tobramycin was *mexXY*, an efflux pump for aminoglycoside resistance indicating that tobramycin treatment further exacerbated resistance. A similar study evaluated the influence of ampicillin on *S. aureus* biofilms (Liu et al., 2018), revealing 530 DE genes including upregulation of several resistance pathways and genes encoding adhesion-promoting surface proteins in biofilms formed with vs. without sub-inhibitory ampicillin. The results collectively clarified important mechanisms by which biofilms resist ampicillin and how sub-inhibitory ampicillin enhances biofilm viability and biomass.

Alford et al. (2020) investigated the role of the nitrogen regulator NtrBC in biofilm formation and chronic infections. NtrBC was found to be not only required for swarming and biofilm formation, but also for dissemination to distal organs from a localized subcutaneous abscess in mice. RNA-Seq performed on *ntrB* and *ntrC* deletion mutants showed 790 and 1184 dysregulated genes, respectively, compared to wild-type in swarming conditions, with many involved in nitrogen and carbon metabolism as annotated by the KEGG database. In addition, there was downregulation of genes required for virulence in rat models of *Pseudomonas* lung infection, which matched the *in vivo* data of decreased dissemination. Thus, these results were consistent with the suggestion that NtrBC may be a new target for anti-biofilm therapies.

### Transposon Insertion Sequencing

While most studies rely on mutants with deletions in specific genes to probe their functions in biofilm formation, transposon insertion sequencing (Tn-Seq) offers a high-throughput approach to identify multiple genes required to survive in a specific condition such as in a biofilm (Cain et al., 2020). Tn-Seq begins by creating a library of mutants with each cell carrying a promiscuous transposon inserted randomly into the genome, and in a library of such mutants, the function of each gene is disrupted in multiple mutants. These mutants can be grown and analyzed individually to elucidate the effect of the mutation on biofilm formation (Ueda and Wood, 2009) but a more efficient approach is to pool these mutants together and grow them collectively to determine which survive in different environments. Direct sequencing is performed on transposon-flanking regions to detect all genes with transposon insertions that exist in the population growing in a specific condition compared to a standard growth control. Mutants with a transposon disrupting a gene that is required for fitness in this condition will not grow well and therefore have decreased representation in the sequencing results. For example, if the mutant pool is sequenced from cells grown under planktonic and biofilm conditions, and a transposon-inserted gene is only detected in the planktonic condition, then that gene is required for biofilm formation (Cain et al., 2020). Genes identified to be required for biofilm formation can then be validated by growing the individual mutants. However, one limitation to pooling mutants for Tn-Seq is that genes encoding extracellular enzymes, proteins, matrix components, or autoinducers that are essential for biofilm formation may not be detected, since mutants of those genes can be cross-complemented by the extracellular components synthesized by non-mutants in the population. This method has incorporated new technologies in the last few years, such as sorting individual mutant cells using microfluidics and using inducible promoters to probe the function of essential genes (which cannot be analyzed using traditional Tn-Seq methods as disruption of essential genes results in non-viable mutants) as outlined in a recent review (Cain et al., 2020).

Poulsen et al. (2019) used Tn-Seq to identify 321 core essential genes shared across nine strains of *P. aeruginosa* isolated from human infections and the environment, as well as five different media replicating human sputum, serum, and urine, and conventional LB and M9 media. Considering that regulators of biofilm formation depend on both the stage of biofilm growth and the experimental setting, a similar approach could be performed on different biofilm stages ranging from initial adherence to dispersal, or different *in vitro* and *in vivo* biofilm models to identify “core essential genes” for biofilm formation shared across all settings. The pathways and proteins identified would be attractive targets for novel anti-biofilm agents. In another study, Morgan et al. (2019) found that interfering with biofilm genes in *P. aeruginosa* by deleting *lasR* (QS) or increasing c-di-GMP levels through deletion of the negative regulator *wspF* led, respectively, to decreased and increased biofilm formation, but surprisingly did not affect fitness in a murine chronic wound high-density infection model. Using Tn-Seq, they found 28 mutants that were absent in the chronic wound, with transposons in genes involved in anaerobic growth and metabolic functions, indicating their possible role in wound fitness. Fitness defects were later validated by growing transposon mutants individually. Thus, the ability to combat stressors in high-density populations is critical for maintaining a chronic infection, and forming biofilms does not appear to be the only way that bacteria can survive in chronic wounds, which has implications on how to approach developing therapies for chronic wounds.

Tn-Seq was also used to investigate the formation of persister cells that make biofilms difficult to eradicate. Cameron et al. (2018) generated 4,411 transposon mutants of *P. aeruginosa* and found 137 genes were needed for survival after fluoroquinolone treatment using Tn-Seq. They focused on *carB*, a subunit of the carbamoyl phosphate synthetase for pyrimidine and arginine synthesis, which was found to have the lowest survival rate when disrupted. The *carB* transposon mutant had increased intracellular ATP accumulation, and treatment with arsenate to reduce ATP levels restored antibiotic resistance in this strain. Thus, an agent that inhibits this synthetase, interferes with pyrimidine synthesis, or increases ATP levels would represent a novel method to prevent the formation of persister cells.

### Proteomics

Proteomics can provide additional information on actual protein expression (which is not always coordinated with transcription due to post-transcriptional regulatory/modification mechanisms). While traditionally identified by gel electrophoresis, limitations in detection and quantification have led to the increasing popularity of liquid chromatography/mass spectrometry (LC-MS) methods that can analyze >80% of the proteome (Khemiri et al., 2016). Proteomics can also be done on “sub-proteomes” through specific extraction protocols that analyze proteins in the extracellular matrix (Gallaher et al., 2006), cell wall (Calvo et al., 2005), and bacterial surface (Solis et al., 2014), providing a level of functional detail that is not captured through genetic analyses. For example, to characterize the surface proteins expressed by *S. aureus*, cell shaving proteomics was performed by using proteases to selectively cleave surface exposed peptide epitopes, which were separated using LC-MS and matched to the original protein for identification (Solis et al., 2014). Characterizing matrix proteins can be accomplished by centrifugation and filtration of biofilms to eliminate cells from the biofilm matrix, followed by proteomic analysis (Couto et al., 2015). Furthermore, identifying temporal production of proteins can be accomplished through bio-orthogonal non-canonical amino acid tagging (BONCAT), in which azidohomoalanine (a methionine analog) is added to cultures and incorporated into newly formed proteins. Proteins containing azidohomoalanine can then be enriched for and characterized by LC-MS (Rothenberg et al., 2018). BONCAT has also recently been performed to identify metabolically active bacteria in cystic fibrosis microbiota (Valentini et al., 2020).

A recent study by Suryaletha et al. (2019) leveraged proteomics to identify proteins only expressed in biofilms when compared to planktonic growth of *Enterococcus faecalis*. GO and KEGG functional enrichment found enhanced production of proteins involved in glycolysis, the LuxS QS system, rhamnopolysaccharide synthesis, and arginine metabolism in biofilm growth, all of which would represent biofilm-selective targets for *Enterococcus* (Suryaletha et al., 2019). Similar studies were done comparing *Haemophilus influenzae* and *Mycobacterium tuberculosis* biofilm and planktonic forms to identify anti-biofilm protein targets (Gallaher et al., 2006; Wang et al., 2019). Erdmann et al. (2019) also aimed to uncover a “core proteome,” much like a core essential genome discussed in the above section, through proteomics analyses of 27 clinical isolates of *P. aeruginosa* grown as biofilms or planktonic suspensions. Interestingly, proteomes from these clinical isolates were similar to each other during planktonic growth, but much more divergent in biofilms despite being grown under the same biofilm conditions. While no protein was selectively dysregulated in the biofilms of all isolates, 141 proteins were differentially expressed in at least 50% of the isolates. Functional enrichment showed increased expression of proteins involved in iron metabolism, fatty acid biosynthesis, and outer membrane protein synthesis, and decreased expression of proteins involved in translation, consistent with *in vivo* proteomic data of *P. aeruginosa* in cystic fibrosis patients (Wu et al., 2019). The proteome diversity across these isolates does not favor a “universal” *P. aeruginosa* biofilm-specific protein, although this might have reflected limited resolution, and argues that an anti-biofilm agent would likely need to target multiple effector proteins in order to have an effect on multiple *P. aeruginosa* isolates.

Finally, meta-proteomics provides a fascinating new area of proteomic research to uncover proteins required for multi-species populations. Most recently, this has been done on a community of four soil bacteria (*Stenotrophomonas rhizophila*, *Xanthomonas retroflexus*, *Microbacterium oxydans*, and *Paenibacillus amylolyticus*) that exhibited enhanced biofilm formation when co-cultivated compared to single species. Meta-proteomics identified the abundance of proteins for each species in key metabolic and energy pathways, such as amino acid metabolism and fermentation, that did not occur in single species communities, implicating both competitive and cooperative mechanisms of survival (Herschend et al., 2017). This technology may soon be applied to other multispecies biofilms, such as those found in healthcare or dental settings.

### Metabolomics

Metabolomics analyzes differential production of small molecule metabolites and metabolism intermediates (e.g., carbohydrates, nucleotides, and amino acids). Bacterial populations are lysed, and the contents undergo either liquid, gas, or ion chromatography to separate the various metabolites by size or charge. This is followed by mass or nuclear magnetic resonance spectrometry of each of the separated fraction to identify metabolites. Metabolomics offers a snapshot of the functional changes that result from the transcriptomic and proteomic changes measured by the above methods. Furthermore, the analysis of metabolites that are consumed and secreted can be used to predict biofilm activity (Beale et al., 2013). Several metabolomic studies comparing planktonic and biofilm-associated bacteria have been undertaken (Yeom et al., 2013; Hasan et al., 2015; Wong et al., 2015; Harrison et al., 2019). For example, in *S. aureus*, arginine metabolites were found to be downregulated in biofilms when compared to planktonic samples, suggesting their consumption in the urea pathway (which uses arginine and arginine metabolites) to maintain pH balance in the biofilm environment (Stipetic et al., 2016). This is consistent with transcriptomic data showing upregulation of urea cycle proteins in biofilms (Resch et al., 2005). Thus, targeting the urea cycle might be a novel way of interfering with *S. aureus* biofilm formation.

### Omics Integration

Each of these omics analyses on their own can analyze complex systems as a whole and provide a more comprehensive profile of the complex adaptive biofilm growth state when compared to a single-target reductionist approach. However, integration of these omics data can uncover new connections that might otherwise remain undetected through individual omics. In addition, the detection of dysregulation of a molecule, protein, or pathway by more than one omics method reinforces the observation that it is a key regulator or target for modulation. A variety of different integration methods are currently available, primarily for human studies (Lee et al., 2019; Misra et al., 2019), although similar approaches have been recently considered for lactic acid bacteria (O’Donnell et al., 2020).

One approach is simply to create a protein-protein interaction network by inputting lists of DE genes and proteins, such as through the web-based platform NetworkAnalyst, which can annotate data from bacterial species such as *P. aeruginosa* and *E. coli* (Xia et al., 2015). Metabolomic data can be linked to this network through protein-metabolite interactors identified by MetaBridge (Hinshaw et al., 2018). Functional enrichment of these combined networks can highlight biologically relevant pathways derived from the combination of these omics analyses.

More complex integrative approaches are available such as through the R package mixOmic (Rohart et al., 2017). One of the issues with multi-omics data is the high dimensionality of each data source, since there can be thousands of genes and proteins in each omics data set. mixOmics can perform dimensional reduction by combining related factors in each dataset and highlighting the factors that provide the largest source of variation, creating a single factor matrix from multiple data matrices that can then be used for functional enrichment (Rohart et al., 2017).

With single-cell omics employed more frequently in other fields, single-cell technologies may be a potential future direction to analyze the heterogeneity of biofilms (Ma et al., 2019). For example, bulk RNA-Seq employed in most studies measures only the average gene expression; single-cell RNA-Seq for biofilms would measure gene expression of each cell, capturing the heterogeneity and pseudo-differentiation of biofilm cells. Regardless of the analysis method, whether it is single-omics or multi-omics, the data generated by these approaches have deepened understanding of biofilm formation and identified novel targets for modulating biofilm growth. The next logical step is to identify novel therapeutics that can act on these targets, which can be accomplished using *in silico* screens and models.

## *In silico* Screens and Models for Identifying Novel Anti-Biofilm Agents

The above omics experiments indicate that several hundred proteins are apparently required for biofilm formation, each representing attractive candidates to modulate or inhibit using small molecules. While screening these compounds has been traditionally performed experimentally, computational (*in silico*) approaches represent an intriguing and potentially time-saving tool for designing and screening anti-biofilm agents. The appeal of deriving and screening agents computationally is multifold, including the ability to learn specific molecular properties associated with biofilm eradication and improved decision making in selecting candidate agents for validation (Vamathevan et al., 2019). Furthermore, *in silico* approaches are facilitated by increasing processing speeds and most importantly, large databases of putative molecules and their specific properties. *In silico* approaches that have been proposed include molecular docking screens, quantitative structure-activity relationship (QSAR) based modeling, and machine learning.

Virtual molecular docking screens rely on estimating the interaction between the 3D structures of targetable bacterial receptors and known ligands or small molecules. This approach was recently given a huge boost with major enhancements in the ability to computationally predict protein structures based on the primary sequence with amazing accuracy (Service, 2020). Interactions between specific receptor-target pairs are empirically scored by estimated hydrogen bonding, and electrostatic and hydrophobic interactions, with high scoring candidates representing novel targets (Schneider and Fechner, 2005; Dos Santos et al., 2018; Guedes et al., 2018). Of more recent interest are machine learning and QSAR methods, a suite of techniques enabling efficient screening and selection of agents in specific contexts, such as peptides targeting *E. coli* biofilms. These modeling frameworks have been reviewed previously (Tamay-Cach et al., 2016; Cardoso et al., 2019), largely by describing their general use in discovering antimicrobials. Furthermore, these two methods may be complementary, since virtual screens may generate compounds that can then be used to train machine learning models. In this section, we discuss virtual screening methods and machine learning methods for deriving candidate anti-biofilm agents and provide several examples of how they have been implemented.

### Virtual Screening

Virtual molecular docking screens permit thousands of compounds in databases to be screened for binding against (and potentially modulating) protein targets identified by omics studies. The starting point is knowledge of the actual (crystallization or NMR derived) or predicted structure, and especially the active sites of the protein in question. Docking algorithms employ algorithms to iterate through possible binding conformations, which are typically optimized to maximize molecular interactions and minimize binding energy to a target protein. Several molecular docking tools have been published and also exist on web applications allowing easier accessibility for researchers, including HADDOCK, UCSF DOCK, and MTiOpenScreen (Allen et al., 2015; Labbé et al., 2015; van Zundert et al., 2016).

In the context of anti-biofilm agents, there are several recent studies that have described the use of molecular docking to screen molecules targeting QS proteins, diguanylate cyclases, (p)ppGpp synthetases, and other regulatory proteins (Fernicola et al., 2016; Kalia et al., 2017; Tiwari et al., 2018; Alves-Barroco et al., 2019; Ding et al., 2019; Mellini et al., 2019; Hall et al., 2020). More recently, Mellini et al. (2019) screened >1000 FDA-approved drugs for binding to PqsR, a previously crystallized protein involved in QS in *P. aeruginosa*. Exclusively screening FDA-approved drugs, an approach known as “drug repurposing,” expedites clinical translation since the drugs’ attributes in humans including toxicities are known. The authors identified five drugs that bind to PqsR with high affinity, and then validated these using *in vitro* biofilm and swarming motility assays. Similarly, Alves-Barroco et al. (2019) used the ZINC database to screen molecules against biofilm-regulatory protein BrpA from the bovine mastitis pathogen *Streptococcus dysgalactiae* subsp. *dysgalactiae*, employing the Auto Dock Vina docking tool (Trott and Olson, 2010; Alves-Barroco et al., 2019). Because the crystallized structure of BrpA was unavailable, a BrpA homolog was submitted to Protein BLAST to identify structural templates. Nevertheless, the resultant molecules included ones with effective, albeit somewhat weak, anti-biofilm activity, providing a template for optimization. As more bacterial proteins are crystallized and solved, protein structural prediction algorithms become more accurate, databases of active anti-biofilm agents grow, and accessibility of docking software improves, the potential of virtual screening can increase dramatically and may soon become a standard technique employed after discovering a new protein target to uncover novel modulators of specific targets.

### Machine Learning

**FIGURE 3:**
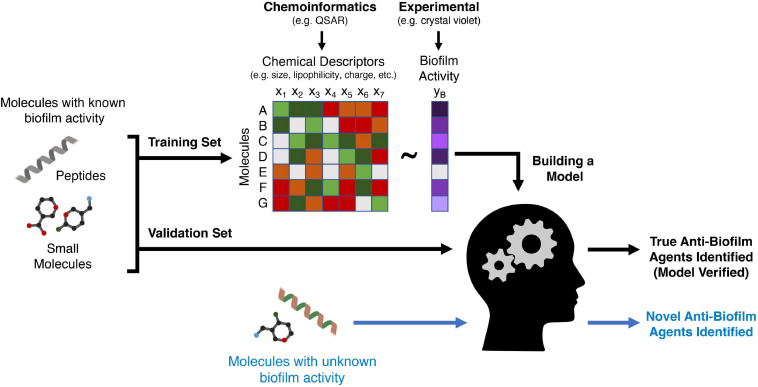
A schematic of how machine learning can be used to discover new anti-biofilm agents. A training set comprising peptides or small molecules with known anti-biofilm activity (validated experimentally) are used to train a machine learning algorithm. Chemical features of these molecules (such as size or lipophilicity) can be generated using chemoinformatics methods such as QSAR. The algorithm then creates a mathematical relationship between a variety of features of each molecule and the anti-biofilm activity of the molecule. A second group of molecules (a validation set) with known anti-biofilm activity is then analyzed using the derived algorithm to ensure that it can accurately classify these molecules as having anti-biofilm activity or not. Finally, molecules for which anti-biofilm activity is not known, are then classified by the algorithm, with the output being potentially novel anti-biofilm agents that must then be validated with *in vitro* or *in vivo* models.

While virtual docking screens rely on accurate 3D structures to predict activity, machine learning is a more flexible approach that focuses on the properties of the molecule itself rather than the target to infer anti-biofilm activity. Machine learning is a set of efficient and powerful statistical methods used to make predictions in various contexts, including the prediction of novel antimicrobial agents specifically targeted to biofilm infections. Generally, an algorithm is trained using large relevant datasets (training sets) in order to learn a relationship between the features describing the data and the prediction task at hand. In the context of small molecule anti-biofilm agents, these features (also termed molecular descriptors) include steric size, lipophilicity, and 3D structure, but there are hundreds of physical-chemical parameters that can be utilized (Figure 3). The goal of a good machine learning model is generalizability to unseen examples; thus, the accuracy of predictions is typically assessed on examples not used for training (a validation set). Several algorithms have been developed to extract complex linear and non-linear relationships and formulate them into predictive models, including Logistic Regression, Random Forest, Support Vector Machines (SVMs), and Neural Networks (Noble, 2006; LeCun et al., 2015).

In order to implement any machine learning technique to identify novel anti-biofilm agents, the prediction task must be established. The prediction task is often as simple as classifying an agent as “anti-biofilm” and “non-anti-biofilm,” representing positive and negative training examples, respectively. Therefore, a set of agents, including small molecules, peptides, or existing antibiotic backbone structures, must be gathered and associated with a particular activity. Several databases comprising small molecules and peptides exist for these purposes, including SwissProt, PubChem, the Antimicrobial Peptide Database (APD), the Biofilm-active AMPs database (BaAMPS), aBiofilm, and the Data Repository of Antimicrobial Peptides (DRAMP) (Di Luca et al., 2015; Wang et al., 2016; Rajput et al., 2018; Kang et al., 2019; Kim S. et al., 2019; UniProt Consortium, 2019). BaAMPS was created to provide researchers a source of peptides to train machine learning models with antibiofilm activity (Di Luca et al., 2015). Gupta et al. (2016) used the BaAMPS database to select 178 anti-biofilm peptides for training an SVM model, whereas the non-anti-biofilm set was composed of randomly generated peptides from all SwissProt database sequences. Similarly, Sharma et al. (2016) used the BaAMPS database to select 80 anti-biofilm peptides to train an SVM model, while their non-anti-biofilm set included only QS peptides with no anti-biofilm/antimicrobial effects. However, while the model accurately predicted known anti-biofilm peptides, its ability to predict unknowns was not verified. Moreover, it is important to note that such validations assume a reproducible standardized assay for evaluation, with *in vitro* MIC compared to biofilm inhibitory concentration (BIC), and/or minimal biofilm inhibitory concentrations (MBIC) (Haney et al., 2018b). This has implications when implementing and comparing various machine learning models across studies, since the exact definition of anti-biofilm may differ.

Beyond the specific assays or mechanisms used to define anti-biofilm activity, an agent’s molecular type (peptide, small molecule, lipid, etc.) and the specific bacterial species are also components of the prediction task. Whereas most machine learning pipelines used to predict anti-biofilm activity have been peptide based, smaller natural and synthetically derived molecules can also be modeled using machine learning. For example, machine learning models that predicted the anti-biofilm activity of naturally occurring essential oils were successfully implemented (Artini et al., 2018; Patsilinakos et al., 2019). Interestingly, Patsilinakos et al. (2019) assayed essential oils for two strains of *S. aureus* and two strains of *S. epidermidis* and used the results to train separate models for each strain. The anti-biofilm activity of each essential oil varied greatly for each strain, highlighting the value in training strain-specific machine learning models (Patsilinakos et al., 2019). Accordingly, the context in which machine learning models are trained can become quite specific, which must be considered when establishing the predictive scope and applicability of a machine learning model, and the need for drugs with broader spectra of activity.

To train a machine learning model, anti-biofilm agents of interest must have accurate numerical representations of physicochemical and 3D properties in the form of features/descriptors. The obvious assumption is that molecules with similar activities have similar physicochemical properties, whereby the approximate relationship between properties and activity are learned during the training of a machine learning model. Extracting features from molecules is an established discipline in itself, referred to as chemoinformatics. QSAR was an early chemoinformatics framework for extracting numerical descriptors from molecules, followed by training a simple machine learning algorithm (Cherkasov et al., 2014; Mitchell, 2014). There are a variety of diverse QSAR categories composed of hundreds of different descriptors extensively curated since inception, including topological, functional groups, and geometric (Danishuddin and Khan, 2016). Many examples of commercial and freely available software exist to extract feature descriptors for a variety of molecules (Sawada et al., 2014).

In this context, Haney et al. (2018a) trained a logistic regression model using seven QSAR descriptors to identify anti-biofilm peptides against methicillin resistant *S. aureus* (MRSA), derived from the widely studied 1018 peptide. The model was validated against a set of 100,000 semi-random peptides and predicted anti-biofilm potential of a previously undescribed peptide 3002. *In vitro* validation showed that peptide 3002 had 8-fold enhanced anti-biofilm potency against MRSA biofilms when compared to 1018, and equivalent activity in a mouse abscess model. Thousands of descriptors were extracted from each peptide in the training set; however, computational prediction reduced this to a set of seven core descriptors that were ultimately used to train machine learning models. This strategy was employed to increase a machine learning model’s generalizability by removing redundant descriptors and preventing overfitting.

Chemical graphs and fingerprints, which capture the atomic structure and connectivity of the molecule, are other approaches for representing molecules numerically for application in machine learning (Lo et al., 2018). These representations are typically appropriate for small molecules rather than peptides with complex and/or flexible secondary structures. Srivastava et al. (2020) trained a hybrid random forest model based on QSAR type descriptors and chemical fingerprints to identify potential anti-biofilm molecules. The authors extracted a 10,208-unit chemical fingerprint, which they combined with the QSAR descriptors to generate a hybrid classifier. Neural networks are a class of machine learning models that mimic the operations of neurons in the brain. Specifically, they allow the models to both learn features through hidden layers and then use them to perform the prediction task. Stokes et al. (2020) used a directed message passing deep neural network (Yang et al., 2019) to learn a type of chemical fingerprint based on the graph structure. Although the authors did not aim to discover an anti-biofilm agent, they predicted and validated the potential use of the antibiotic halicin for use against *E. coli* infections. Furthermore, this study represents a use of neural networks and feature learning which can be applied to identify novel anti-biofilm agents. In this regard, similar neural network approaches have been used to derive enhanced 9-amino-acid, broad-spectrum antimicrobial peptides, by relating descriptors to activity (Cherkasov et al., 2009). It is often stated in machine learning “garbage in, garbage out,” meaning poor quality input data results in poor predictions. Accordingly, a large training set such as the one used by Cherkasov et al. (2009), a diverse set of accurately estimated features and descriptors, and robust modeling techniques are essential to predict novel anti-biofilm agents.

To assess the predictive ability of a trained machine learning model, a test or validation set is employed. This set includes agents where the anti-biofilm activity is known, thus the model’s predictions can be compared for accuracy. Practically speaking, cross validation is often employed in which a subset of, for example, 80-90% of molecules with known activity is used for testing and the remainder for validation, and this is repeated iteratively using a different subset of molecules for validation. Finally, novel agents predicted as having anti-biofilm activity must be confirmed with *in vitro* or *in vivo* experiments as described in the next section. The application of machine learning to predict anti-biofilm activity has often proven successful, as shown in the presented studies and broader studies identifying antimicrobial agents. Future directions in this field will include expanding databases of anti-biofilm agents and their respective potencies, as well as determining drug parameters such as pharmacokinetics/pharmacodynamics, bioavailability, and toxicity, to advance commercialization and provide an extensive repository of training and validation molecules for various prediction tasks. Whereas most studies have predicted anti-biofilm activity in a binary fashion, directly predicting potency may also generate better candidates. Expanding these resources will immensely benefit the community in building robust and generalizable machine learning models for novel anti-biofilm agents.

## Models for Assessing Novel Anti-Biofilm Agents

**FIGURE 4:**
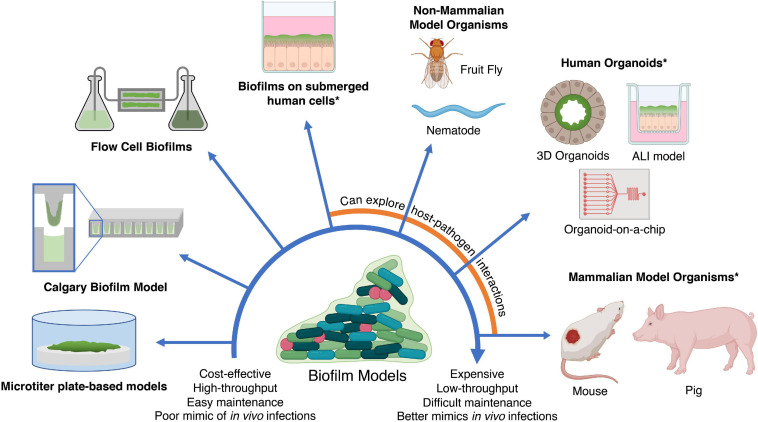
*In vitro* and *in vivo* models for testing anti-biofilm agents. Simplistic models are easier to maintain and cost-effective; however, they fail to replicate *in vivo* infections as accurately as more expensive models such as *in vivo* animal models and human organoids. In addition, the use of human cells and mammalian models (indicated by asterisks) may require additional ethics approval. ALI, air-liquid interface. Created with BioRender.com.

The *in silico* methods described above can generate potential candidates. However, validation is required in accurate biofilm models to assess their anti-biofilm activity and clinical potential. An ideal biofilm model should provide high-throughput testing of multiple compounds against multiple species, be easily manipulatable, and to some extent resemble biofilms found in human infections and on abiotic surfaces such as medical devices. However, while no biofilm model exists that satisfies all three conditions, this section provides an overview and discussion of the advantages and limitations of current *in vitro* and *in vivo* based biofilm models to facilitate a decision on which biofilm model to use in different situations (Figure 4). These biofilm models can also be used for experiments to generate omics data as discussed earlier.

### *In vitro* Biofilm Models

The purpose of an *in vitro* biofilm model is two-fold: (i) to provide a method of assessing the relative activities of a group of compounds and relating these to other compounds in the literature, and (ii) assessing the probability that compounds will work against biofilms in a relevant circumstance (e.g., a biofilm infection in a patient). Such *in vitro* models can be generally classified into closed, open, and tissue culture-based model systems. Closed or static models have no influx or efflux of nutrients, while dead cells, waste, and signaling byproducts will build up (Lebeaux et al., 2013). In addition, closed systems do not always reflect conditions found under some circumstances *in vivo*, such as shear stress from constant movement of liquids in the bloodstream or in medical devices such as catheters (Lebeaux et al., 2013). This issue might be somewhat overstated however, since biofilms in the body often occur on tissues (e.g., wounds, burns, skin, tissues, prosthetic joints, sinuses, bones, etc.), where it can be argued that there is minimal flow of liquids. Microtiter plate-based biofilm systems are classic examples of the closed model. Following on from the popular methods for determining minimal inhibitory concentration (MIC) for antimicrobials against planktonic bacteria (Wiegand et al., 2008), we have recently proposed a standardized method for assessing anti-biofilm activity (Haney et al., in press).

Microtiter plate biofilm assays are one of the most widely used *in vitro* model systems, where biofilms are grown on the bottom or the walls of a microtiter plate or on materials (e.g., microscope slides, silicone, titanium, and hydroxyapatite disks) placed within a microtiter plate (Vandecandelaere et al., 2016). They represent a relatively cheap and user-friendly system, with parallels to MIC assays, that can be easily used as a high-throughput screen, require only a small volume of reagents, provide researchers easy control over growing conditions (media type, temperature, humidity, and presence/absence of stress signals), and enable examination of various stages of biofilm development (Coenye and Nelis, 2010; Vandecandelaere et al., 2016). Numerous studies have used the microtiter biofilm system to understand biofilm formation on various biomedical materials and surfaces (Chin et al., 2006; Imamura et al., 2008; Silva et al., 2010; El-Ganiny et al., 2017; Wang et al., 2018), elucidate biofilm adaptation under different growth conditions (Strempel et al., 2017), screen for biofilm-deficient mutants (Tu Quoc et al., 2007; Okshevsky et al., 2018; Willett et al., 2019), and determine the efficacy of antimicrobial and antibiofilm therapies (Torres et al., 2018; Wang et al., 2018; Zhong et al., 2019). There are a wide range of relatively simple techniques to quantify the amount of biofilm in microtiter systems, including assessing colony forming unit (CFU) counts and staining of adhered bacteria. Staining methods such as crystal violet staining can be used to evaluate the total biomass, while tetrazolium-based dyes, resazurin, the BacTiter-Glo assay (which quantifies ATP production), or propidium iodide can be used to determine the residual number of metabolically active cells (Peeters et al., 2008; Vandecandelaere et al., 2016; Maiden et al., 2018; Wang et al., 2018).

There are several limitations to the aforementioned microtiter methods since they are closed models. The most profound of these is that many microtiter protocols involve adding the anti-biofilm agents along with planktonic bacteria during inoculation; therefore, it is difficult to differentiate between inhibition of planktonic growth, inhibition of the initial stages of biofilm development, killing of organisms in the biofilm growth state, or eradication/dispersal of mature biofilms (Haney et al., 2018b). Second, some staining methods make it difficult to discriminate between dead and live cells, since the most popular staining procedure, using crystal violet, stains the total biomass including matrix and dead cells (Peeters et al., 2008). To overcome these limitations, Haney et al. (2018b) described a simple, cost-effective, and reproducible procedure to assess the biofilm inhibition (addition at the time of bacterial addition) and eradication (delayed addition) abilities of antibiotics and anti-biofilm peptides, using common and relatively inexpensive materials. This high-throughput workflow combines a 96-well microtiter plate with crystal violet or tetrazolium chloride dye staining (Haney et al., 2018b). It should be mentioned that the medium leading to optimal biofilms growth varies substantially between species, so this is one parameter that needs to be optimized in such microtiter systems.

Ceri et al. (1999) developed a rapid system to evaluate the biofilm eradication ability of certain compounds, called the Calgary Biofilm Device. This system involves a specialized top lid with pegs that fits over a conventional 96 well microtiter plate. Biofilm is first grown on the pegs, then the top lid is transferred into a second plate containing the compound of interest. Upon incubation, the amount of residual biofilm can be determined either through CFU count, optical density measurement, or microscopic techniques (Moskowitz et al., 2004). This method allows for the differentiation of biofilm from sedimented dead cells, thus also ascertaining biofilm eradication versus inhibition activity of compounds.

As mentioned above, biofilms in closed systems do not reflect situations where biofilms are exposed to the complex flow network of the circulatory, urinary, digestive, and respiratory systems, nor do they reflect biofilms in medical devices exposed to flowing liquid, such as catheters and intravenous lines (Waters et al., 2014). To understand the biofilm mechanisms and susceptibilities under conditions with constant flow, an open system should be used. However, such systems are considerably more technically complex.

Open systems have a constant flow of fresh medium, while wastes, signaling byproducts, and planktonic cells are constantly washed away, mimicking certain environments in human hosts and medical devices (Lebeaux et al., 2013; Azeredo et al., 2017). The environment of an open system can be controlled and adjusted by the researcher at any time during the experiment. For example, the flow and type of medium can be adjusted to create shear forces and nutrient composition that more closely reflect *in vivo* conditions (although shear forces vary depending on the clinical situation and are not always precisely known). This allows the study of physical and chemical resistance of biofilms. Flow cells are the most widely used open system, consisting of a series of growth chambers that are separately connected to a multichannel peristaltic pump, allowing the influx of fresh media and efflux of waste (Crusz et al., 2012). This system can be coupled with fluorescence or confocal laser scanning microscopy, enabling one to non-invasively visualize the development of a biofilm in real time and reconstruct 3D images of the biofilm structure, which cannot be done with microtiter assay methods (Heydorn et al., 2000; Millar et al., 2001; Mueller et al., 2006; Pamp et al., 2009). For example, Pihl et al. (2013) used flow cells and confocal laser scanning microscopy to model biofilm growth in catheters to show that rhamnolipids in the supernatant of *P. aeruginosa* play a role in reducing adherence and inducing dispersal of *S. epidermidis* to serum coated catheter surfaces. However, the construction and operation of a flow cell can be challenging and requires some expertise (Crusz et al., 2012) and it is less amenable to high-throughput analyses when compared to microtiter assays. Newer microfluidic models, also a type of open model, may alleviate this problem and are discussed below.

Although bacterial *in vitro* models are valuable tools for studying biofilms and screening for potential anti-biofilm agents under controlled conditions, there are some limitations. It is essential to understand the impact of the host-microbe interaction (e.g., nutrient composition, host immunity, and stress factors) and recapitulate the timescale and complex physical and chemical environments bacteria may experience *in vivo*; all of these are absent in these *in vitro* models. These factors can greatly impact bacterial virulence and biofilm formation, which can hinder interpretations of antimicrobial efficacy (Palmer et al., 2007; Kolpen et al., 2010; Pearce et al., 2018; Pulkkinen et al., 2018). In addition, some bacteria found in *in vivo* biofilms are simply not culturable *in vitro* (Li et al., 2014). At least 92 species of bacteria were found in human dental plaque samples, of which eight were uncultivable but were associated with early stages of biofilm formation (Heller et al., 2016). Thus, it is important to also consider *in vivo* models, not only to validate *in vitro* results, but also to factor in the dynamic host-microbe relationships to form more biologically accurate biofilms for testing novel anti-biofilm agents.

### *In vivo* Biofilm Models

*In vivo* biofilm models involve the use of living organisms, including both mammals and non-mammals (Lebeaux et al., 2013). While conventional mammalian models (e.g., mice, rats, and rabbits) are widely used to study *in vivo* biofilms and anti-biofilm agents, screening large numbers of potential antimicrobial candidates is costly, laborious, and ethically prohibited. To overcome these limitations, non-mammalian models, such as the nematode *Caenorhabditis elegans* (Millet and Ewbank, 2004) and fruit fly *Drosophila melanogaster* (Ferrandon et al., 2007), can be used to conduct initial candidate screening before moving to mammalian models.

Non-mammalian models have several advantages over mammalian models. First, such organisms have unique anatomy, but possess some similar immune responses when compared to mammals, and generally allow for easier monitoring of disease progression and the effects of antimicrobial agents (Antoshechkin and Sternberg, 2007; Lemaitre and Hoffmann, 2007; Ryu et al., 2010). Both *C. elegans* and *D. melanogaster* larvae are transparent, which allows for non-invasive monitoring of fluorescently tagged bacteria, host genes, or proteins in real-time (Bell et al., 2009; Kong et al., 2016). *C. elegans* also possess at least three of the innate immunity signaling pathways (p38 mitogen-activated protein kinase pathway, insulin/growth factor-1 pathway, and the transforming growth factor-β pathway) found in mammals (Mallo et al., 2002; Garsin et al., 2003; Troemel et al., 2006). *D. melanogaster* possess host defenses mechanisms such as Toll-like receptor pathways, host defense peptides, and reactive oxygen species (Dimarcq et al., 1994; Agaisse and Perrimon, 2004; Lemaitre and Hoffmann, 2007; Bell et al., 2009). Second, when compared to mammalian models, these non-mammalian models are more easily genetically manipulated from the perspective of both protocols and ethics, enabling investigation of the roles of host factors including immunity in biofilm formation (D’Argenio et al., 2001; Antoshechkin and Sternberg, 2007; Chakrabarti et al., 2012). Third, non-mammalian organisms are highly fertile with short reproduction times and easy maintenance (Antoshechkin and Sternberg, 2007; Jennings, 2011), making high-throughput candidate screens possible (Ewbank, 2002; Squiban and Kurz, 2011; Conery et al., 2014). However, there are profound differences in physiology, many immune responses, circulation, pharmacokinetics, and prospective delivery methods, and data gleaned are not useful in formal drug development. In addition, the short lifespan of *C. elegans* and *D. melanogaster* makes these models difficult for representing chronic infections, and the body temperatures of *C. elegans* (16-25°C) are not optimal for growth of many pathogens and do not reflect that of mammals (Kong et al., 2016). Thus, the use of mammalian models, which have a more complex immune system, relatively longer lifespan, and closer evolutionary relationship to humans, is required.

Mammalian *in vivo* models are indispensable tools to mimic human biofilm infection in the context of host-microbe interactions and to assess antimicrobial therapies before clinical trials (Festing, 2004). There are many well-established mouse (*Mus musculus*) models for biofilm-related diseases, including cystic fibrosis and chronic obstructive pulmonary disease-associated infections, urinary tract infections, intestinal infection, chronic skin/wound infections, chronic rhinosinusitis, and periodontitis (Coenye and Nelis, 2010). The small size, ease of handling and housing, short gestation period, and high reproductive rate of mice make such models attractive when compared to other mammalian models (Rumbaugh and Carty, 2011; Masopust et al., 2017). A recent study used a very simple cutaneous infection mouse model to demonstrate the efficacy of synthetic cationic peptides IDR-1018 and DJK-5, and their synergy with conventional antibiotics in all ESKAPE pathogens, as well as their relationships to the stringent response (Pletzer et al., 2017, 2018). Both peptides were effective in reducing abscess size and bacterial load, and showed synergy with several different antibiotics, in part through decreased (p)ppGpp synthesis due to *spoT* down-regulation. This abscess model can be used to evaluate other peptides and anti-biofilm agents for chronic wounds.

In addition, a large number of inbred mouse strains (which are genetically uniform to enhance reproducibility), outbred strains (which better represent genetic diversity in human hosts), and genetically modified strains are commercially available and well characterized (Thomas and Capecchi, 1987; Svenson et al., 2012; Masopust et al., 2017). Genetically modified strains allow investigators to induce immunodeficiency, humanize the immune system, and knock in/out specific genes to create phenotypes similar to certain human diseases (Criswell and Sack, 1990; Masopust et al., 2017; Gurumurthy and Lloyd, 2019). For example, a cystic fibrosis transmembrane conductance regulator (CFTR) knockout mouse model was used to study cystic fibrosis and the QS inhibiting effects of azithromycin against *P. aeruginosa* biofilms (Hoffmann et al., 2007).

Another mammalian model that more closely resembles humans than do mice, is the pig (*Sus scrofa domesticus*), especially in terms of their anatomy and immune system (Dawson, 2011; Meurens et al., 2012). Due to the close resemblance of porcine skin to human skin in terms of structure, immune responses, and the process of wound healing, the porcine model has been deemed the most relevant preclinical model of skin wound healing by the Wound Healing Society (Sullivan et al., 2001; Gordillo et al., 2013). Gloag et al. (2019) used the porcine skin biofilm model to identify hyperbiofilm strain variants of *P. aeruginosa*, which were found to have mutations in the Wsp pathway (a chemosensory pathway involved in c-di-GMP regulation) and resistance to prophages in the wound. There are also recent developments to create *ex vivo* porcine skin models for use as a surrogate for live pigs, to improve ease of use and allow for high-throughput setups (Alves et al., 2018).

Although *in vivo* animal models are invaluable to investigate host-pathogen interactions, there are also some limitations. Interspecies differences still contribute to discrepancies in pharmacokinetic profiles, safety, and efficacy of therapeutic candidates between animal models and humans (Jansen et al., 2020). The growing awareness of animal welfare and related ethical issues encourages researchers to follow the 3Rs: Replace the use of animal models, Reduce the number of animals required for each experiment, and Refine experimental techniques to minimize animal suffering and improve animal welfare (Bailey and Balls, 2019; Hubrecht and Carter, 2019). To satisfy the first “R,” an alternative model that can investigate host-pathogen interactions in the context of anti-biofilm therapies is tissue culture-based *in vitro* models.

### Tissue Culture-Based Biofilm Models

Tissue culture-based are co-cultures of bacterial and human cells. Conventionally, submerged models are used, where a biofilm is grown over a monolayer of host cells submerged in medium (Coenye and Nelis, 2010). Compared to *in vivo* models, submerged monolayer models are cheaper, easier to manipulate, highly reproducible, and amenable to high-throughput screening, while still enabling investigation of host-pathogen interaction; however, they lack cell type complexity, commensal flora, nutrient gradients, shear forces, and immune components (Coenye and Nelis, 2010). For example, *Mycoplasma pneumoniae* biofilms grown on a monolayer of human bronchial epithelial cells were found to undergo similar architecture development as those grown on glass, but at a slower pace (Feng et al., 2020). However, the presence of complement significantly reduced the growth of *M. pneumoniae* on epithelial cells, suggesting that bacterial growth might be significantly different in a more complex system (Feng et al., 2020). In addition, most submerged models can only mimic acute infections, since culturing bacteria and host cells within a static condition leads to high cytotoxity (Kim et al., 2012). Hence, it is important to use alternative co-culture systems than submerged models that more closely resemble *in vivo* microenvironments.

A recent advance in tissue culture techniques is the development of host organoid systems, which overcome some of the limitations of submerged monolayer models (Barrila et al., 2018). Organoids are self-organized, multicellular structures that resemble miniature organs, and can be derived from immortalized cell lines, primary cells from healthy or diseased donors, induced pluripotent stem cells, embryonic stem cells, neonatal tissue stem cells, or *ex vivo* adult progenitors (Clevers, 2016). In general, organoid systems can be categorized into three main forms, in order of complexity: air-liquid interface (ALI) models, 3D spheroid organoids, and organoid-on-a chip models (Choi et al., 2020).

Air-liquid interface models are grown from a variety of different starting cells and differentiated on permeable filters to form sections of epithelium, with the apical region exposed to air and basal region submerged in medium (Choi et al., 2020). The dual exposure allows maturation of multiple cell types with different functions (e.g., mucin production, cilia movement) similar to those found *in vivo*, and there are ALI models for skin, lung, intestinal, gingival, and urothelial epithelium (Dvorak et al., 2011; Pezzulo et al., 2011; de Breij et al., 2012; Horsley et al., 2018; Brown et al., 2019; George et al., 2019). In addition, the presence of an apical and basal chamber provides a convenient platform for co-culture systems to investigate immune activity against biofilms on epithelium. Rudder et al. (2020) developed a triple co-culture ALI system, with upper respiratory tract epithelial cells, macrophages in the basal chamber, and donor nasal microbiota in the apical chamber, and found that diversity of microbial communities was altered by the addition of macrophages (Rudder et al., 2020). Similarly, a gingival epithelium ALI model studied oral biofilms formed by healthy microflora or microorganisms in gingivitis and periodontitis in the presence of primary peripheral blood mononuclear cells and CD14+ monocytes in the basal chamber (Brown et al., 2019). Recently, a miniaturized 96 well air-liquid interface human small airway epithelial model was developed, allowing ALI models to be used as a high-throughput screening platform (Bluhmki et al., 2020). An analogous skin model was established from N/TERT keratinocytes (Wu et al., 2021; de Breij et al., 2018), which enabled well-structured biofilms to be grown from *P. aeruginosa* and *S. aureus* and allowed for screening of the effects of various antibiofilm peptides and their influence of skin integrity.

A more complex system is 3D spheroid organoids, in which progenitor cells undergo stepwise directed differentiation with defined growth factor cocktails that activate and inhibit specific signaling pathways (Nickerson et al., 2001; Clevers, 2016; Gkatzis et al., 2018). Generally, 3D organoids mimic the *in vivo* architecture, multi-lineage differentiation, and organ development process of the natural epithelium in mammals (Sato and Clevers, 2013; Kim M. et al., 2019). Their enclosed nature can allow growth of bacteria that are unable to be cultured in other *in vitro* systems (Dutta et al., 2017; George et al., 2019). Furthermore, the ability of self-regeneration allows 3D organoids to be maintained and expanded over a long period of time to study chronic infections (Yamamoto et al., 2017; Sachs et al., 2019). Coupling the 3D organoids with microinjection and imaging techniques, Forbester et al. (2015) showed that *Salmonella enterica* serovar Typhimurium was able to invade the epithelial barrier and reside in vacuoles, similar to those found *in vivo* (Forbester et al., 2015).

While 3D organoids and ALIs better replicate *in vivo* conditions than other *in vitro* models, they still lack a dynamic mechanical and biochemical microenvironment with shear force and nutrient gradients as provided by flow cells. Combining these two technologies results in the organoid-on-a-chip model, which is a microfluidics platform where bacteria and/or host cells grow in chambers perfused by microchannels (Kim et al., 2012). By strictly controlling intraluminal fluid flow to mimic peristalsis, Kim et al. (2012) developed a microfluidic model with intestinal epithelium that resembled the structure of intestinal villi and supported growth of *Lactobacillus rhamnosu*s for >1 week without compromising host cell viability. Since only a small volume of cells and reagents are required, organoids-on-a-chip models appear to be a relatively cheaper and faster organoid screening method, although it requires a sophisticated, technically complex, and expensive platform to set up such experiments (Huh et al., 2011). Cells from microfluidic chambers can also be extracted for omics studies such as transcriptomics (Benam et al., 2016). Furthermore, microsensors embedded in the chip allow monitoring of events such as biofilm formation, cell migration, barrier function, protein production, and fluid pressure in real time (Bhatia and Ingber, 2014; Yu et al., 2019; Yuan et al., 2020). Yuan et al. (2020) developed an integrated system, combining gut-on-a-chip with optical coherence tomography, to visualize pathogenic *E. coli* mediated cellular changes in the presence or absence of probiotic protection of *Bifidobacterium breve* in real-time. Similarly, Sidar et al. (2019) incorporated 3D spheroid intestinal organoids with a microfluidics system to study intestinal secretion, absorption, transportation, and co-culture with intestinal microorganisms. Finally, a multifaceted combination of microchambers, microchannels, valves, pumps, and microsensors allows organoid-on-a-chip models to be tailored to specific needs for different experiments (Yu et al., 2019). For example, Ye et al. (2007) utilized a microfluidic device with eight drug gradient generators and parallel cell culture chambers to simultaneously test either eight different molecules or eight different concentrations of one molecule. This system has the potential to be used as a high-throughput screening system for antibiofilm agents. Organoid-on-a-chip models are relatively new, and their versatility makes them perhaps the closest to a biologically accurate model that still allows the ability of higher-throughput testing.

Each model has its own advantages and disadvantages, ranging from cost and ease of use to similarity to *in vivo* biofilms, with the latter point perhaps being the most important for success in developing an anti-biofilm agent that will be effective in humans. Furthermore, each of these models can also be used in conjunction with further omics analyses to better understand the mechanisms of the agents tested. With the advent of new technologies such as organoid-on-a-chip models, the prospects of creating a high-throughput, biologically relevant model for anti-biofilm agent testing are tantalizingly close.

## Conclusion

In this review, we have provided a summary of current biological and computational strategies to develop new anti-biofilm agents. Omics analyses provide a systems biology approach to the complex interwoven processes of biofilm formation and are uncovering many potential protein targets and pathways required for biofilm formation in a variety of species. To find modulators for these targets, high-throughput screening using *in vitro* approaches have been used in the past to test potential modulators; however, with the increased availability of defined bacterial protein structures, recent approaches now more commonly involve an initial virtual screening of large databases of molecules before experimental validation, which is more cost-effective and less labor-intensive. Another approach to identify novel anti-biofilm agents is through machine learning, where a model is trained using a collection of known anti-biofilm and non-anti-biofilm molecules, learns patterns in the features of these molecules, and then applies those patterns to pick out potential anti-biofilm agents from databases. Finally, these new agents must be tested in biologically accurate biofilm models. While *in vitro* approaches such as microtiter assays are the easiest to work with, they poorly resemble actual *in vivo* infections in humans. However, animal models are more difficult to manage both ethically and logistically, and do not accurately resemble human physiology. The rise of organoid models such as relatively simple ALI models and more complex organoid-on-a-chip model can provide an *in vitro* approach that mimics human physiology yet retains the high-throughput characteristic of other *in vitro* models. Advances in this field may eventually result in organoids becoming the optimal model for growing and testing anti-biofilm agents.

Biofilm regulation is a complex process and while we have summarized key more-conserved biofilm regulation processes such as the stringent response, quorum sensing, and c-di-GMP signaling, not all processes have been highlighted here. However, the approaches we describe can equally be applied to motility regulation, small non-coding RNA regulation, and matrix synthesis, as well as new mechanisms discovered by methods outlined in this review. As mentioned before, there are no approved agents specifically targeting biofilms, despite biofilms being the most common form of infection and major reason for antibiotic resistance. We submit that anti-biofilm agents, when they do become introduced in the clinic, will likely be used in conjunction with conventional antibiotics as an “antibiotic sensitizer” by disrupting the biofilm and exposing individual bacteria to antibiotic therapy, or as a prophylactic measure to prevent biofilm formation on medical surfaces or prior to or after surgery. Our hope is that this review has provided a comprehensive introduction, for researchers interested in developing anti-biofilm agents, to the variety of technologies and models used for such an endeavor, as such agents are crucially needed in healthcare.

## Author Contributions

AA, AB, and K-YC contributed to writing and editing this manuscript. AA prepared the figures. RH supervised all authors, edited the manuscript, and provided critical insights and feedback. All authors read and approved the final version of the manuscript.

## Conflict of Interest

RH has invented anti-biofilm peptides, related to those discussed here, and assigned these to his employer the University of British Columbia who filed for patent protection and licensed them to the ABT Innovations Inc., a company owned in part by RH. The remaining authors declare that the research was conducted in the absence of any commercial or financial relationships that could be construed as a potential conflict of interest.
